# Genome survey and evolutionary analysis of 8 *Lamprotula* species: SSR profiling, mitochondrial characterization, and population dynamics inference

**DOI:** 10.1093/dnares/dsaf020

**Published:** 2025-08-09

**Authors:** Min Jiang, Qi Liu, Chao Jiang, Mingfei Zhan, Haibo Wen, Fengyue Shu, Lingli Xie, Tengteng Liu, Chenliang Ren, Wenqiao Tang, Kai Liu

**Affiliations:** Shanghai Universities Key Laboratory of Marine Animal Taxonomy and Evolution, Shanghai Ocean University, Shanghai 201306, China; Key Laboratory of Freshwater Fisheries and Germplasm Resources Utilization, Ministry of Agriculture and Rural Affairs, Freshwater Fisheries Research Center, Chinese Academy of Fishery Sciences, Wuxi 214081, China; College of Fisheries, Southwest University, Chongqing 400715, China; Wuxi Fisheries College, Nanjing Agricultural University, Wuxi 214081, China; Anhui Shuiyun Environmental Protection Co., Ltd., Wuhu 241000, China; Key Laboratory of Freshwater Fisheries and Germplasm Resources Utilization, Ministry of Agriculture and Rural Affairs, Freshwater Fisheries Research Center, Chinese Academy of Fishery Sciences, Wuxi 214081, China; Wuxi Fisheries College, Nanjing Agricultural University, Wuxi 214081, China; Qufu Normal University, Qufu 273165, China; Key Laboratory of Freshwater Fisheries and Germplasm Resources Utilization, Ministry of Agriculture and Rural Affairs, Freshwater Fisheries Research Center, Chinese Academy of Fishery Sciences, Wuxi 214081, China; Key Laboratory of Freshwater Fisheries and Germplasm Resources Utilization, Ministry of Agriculture and Rural Affairs, Freshwater Fisheries Research Center, Chinese Academy of Fishery Sciences, Wuxi 214081, China; School of Ecology and Environment, Anhui Normal University, Wuhu 241000, China; Shanghai Universities Key Laboratory of Marine Animal Taxonomy and Evolution, Shanghai Ocean University, Shanghai 201306, China; Shanghai Universities Key Laboratory of Marine Animal Taxonomy and Evolution, Shanghai Ocean University, Shanghai 201306, China; Key Laboratory of Freshwater Fisheries and Germplasm Resources Utilization, Ministry of Agriculture and Rural Affairs, Freshwater Fisheries Research Center, Chinese Academy of Fishery Sciences, Wuxi 214081, China; Wuxi Fisheries College, Nanjing Agricultural University, Wuxi 214081, China; School of Ecology and Environment, Anhui Normal University, Wuhu 241000, China

**Keywords:** *Lamprotula*, K-mer analysis, mitochondrial genome, phylogenetic analysis, PSMC

## Abstract

Freshwater bivalves are vital to aquatic ecosystems but face severe global threats. Understanding their genomic traits and evolution is crucial for effective conservation. This study conducted whole-genome sequencing on 8 *Lamprotula* species. These 8 species exhibited high genomic complexity, characterized by large genomes (1.89 to 2.65 Gb), high heterozygosity (>0.8), and high repeat content (>60%), estimated by k-mer analysis. Genome assemblies showed that *L. caveata* had the largest genome, while *L. polysticta* had the smallest. Furthermore, the assembled genome sizes of these 8 species exhibited an average increase of 22.58% compared to k-mer analysis estimates, largely due to their high heterozygosity. The mitochondrial genomes of these 8 species ranged in size from 15.69 kb to 17.13 kb, with GC contents varying from 36.36% to 40.77%. Phylogenetic analysis indicated early divergence of *L. leai* and *L. caveata* from the other 6 species. Pairwise Sequentially Markovian Coalescent analysis revealed population bottlenecks over the past million years, with *L. rochechouarti* showing more significant population size fluctuations during the Pleistocene Glacial Epoch. In summary, this study provides comprehensive genomic insights into 8 *Lamprotula* species, highlighting their high genomic complexity and evolutionary divergence, thereby establishing a crucial foundation for future conservation and genetic research efforts.

## 1. Introduction

Freshwater bivalves serve as vital components of aquatic biodiversity and play indispensable roles in freshwater ecosystems.^1,2^ Functioning as key mediators in nutrient cycling and energy flow, they provide essential ecological services, including water purification, habitat engineering, and biological indicators for environmental monitoring.^3–8^ Furthermore, these molluscs represent crucial trophic resources for benthic carnivorous fishes, crustaceans, and rare avian species. Particularly noteworthy is the obligate symbiotic relationship between unionid mussels (*Unionidae*) and bitterling fishes (*Acheilognathinae*), where the gills of freshwater mussels serve as exclusive incubation chambers for bitterling embryos during their reproductive phase.^9^ As a global biodiversity hotspot for freshwater molluscs, China harbours exceptional species richness. However, escalating anthropogenic pressures, including aquatic habitat degradation, water pollution intensification, and destructive harvesting practices, have precipitated severe declines in native mussel populations.^6,10^ Numerous endemic species now face critical endangerment, with some taxa approaching functional extinction in their natural habitats. Freshwater bivalves (order *Unionoida*) are one of the most threatened animal groups in the world.^11^ This alarming trajectory of biodiversity erosion urgently necessitates the implementation of comprehensive conservation strategies, encompassing habitat restoration, germplasm resource preservation, and ecosystem-based management approaches to safeguard these ecologically invaluable organisms.

*Lamprotula* species belongs to the phylum *Mollusca*, class *Bivalvia*, order *Unionoida*, and family *Unionidae*.^12^ These freshwater bivalves are primarily distributed across East Asia, including China, Korea, Vietnam, and Japan, with approximately 26 species recorded worldwide. This taxon exhibits both ecological vulnerability and evolutionary distinctiveness.^10,13^ Most of these species are found in China, many of which are endemic and possess significant economic and ecological value.^14^ Due to environmental changes, river pollution, and overfishing, these species face the threat of population extinction.^15^ Notably, the International Union for Conservation of Nature Red List identifies 17 *Lamprotula* species as critically endangered, vulnerable or data deficient, yet their evolutionary relationships and adaptive genetic mechanisms remain poorly characterized, highlighting the urgent need for integrated conservation strategies and scientific population assessments.^10,16^ Persistent taxonomic controversies significantly impede conservation efforts for *Lamprotula* species. While Wu et al. proposed the division of *Lamprotula* into 2 genera (*Lamprotula* and *Aculamprotula*) based on morphological and anatomical characteristics,^13,17^ subsequent molecular phylogenetic analyses have corroborated the polyphyletic nature of the genus while suggesting alternative subfamilial placements for these clades.^12^ Additionally, the taxonomic status of *L. rochechouarti* requires clarification, as evidenced by conflicting morphological and molecular biological data.^18^ These unresolved issues regarding interspecific divergence, taxonomic delineation, and phylogenetic reconstruction highlight critical knowledge gaps. However, genomic research on these species is limited, posing significant challenges for understanding their genetic characteristics, evolutionary history, and population dynamics.

While genomic resources for marine molluscs have expanded rapidly, freshwater bivalve genomics lags significantly, primarily focussed on biomineralization and pearl formation mechanisms.^19–21^ For *Lamprotula* species, existing studies predominantly centres on resource assessments,^14^ morphology,^22,23^ taxonomy^24^ and phylogeny.^12^ This lack of data hinders our understanding of its evolutionary history and impedes efforts to mine genetic information for broader phylogenetic studies of the sinipercids. Therefore, it is imperative to conduct genome-level analyses of the germplasm resources, genetic background, and evolutionary history of *Lamprotula* to resolve its taxonomic status and support future domestication, breeding, and conservation biology research.

Currently, the advancement of high-throughput sequencing technologies and the continuous reduction in sequencing costs have significantly improved our ability to study the genetic characteristics of aquatic animals.^25^ Genomic survey analysis has proven to be a highly effective and cost-efficient method for rapidly obtaining key genomic characteristics, such as genome size, heterozygosity rate, repeat sequence proportion, and ploidy level, particularly when no reference genome is available.^26,27^ The high-depth clean data generated from these surveys provides an essential foundation for *de novo* genome assembly. Tools like SOAPdenovo2 have shown their effectiveness in generating high-quality draft genomes using paired-end short reads, offering crucial genomic resources for studying genetic traits.^28^ A wide range of well-established and robust bioinformatics tools allow for the accurate characterization of the mitochondrial genome,^29,30^ the inference of evolutionary relationships,^31^ and the estimation of effective population sizes.^32^ Importantly, the genomic characteristics of *Lamprotula* species obtained through genome survey provide a robust foundation for constructing high-quality reference genomes in future studies.

In this study, a comprehensive whole-genome sequencing analysis was conducted on 8 Lamprotula species. Based on high-depth paired-end data, genome characteristics such as genome size, heterozygosity, and repetitive sequence proportion were estimated using K-mer analysis. Draft genomes were obtained through genome assembly, and the mitochondrial genomes were characterized. Simple sequence repeats (SSRs) were also identified, and phylogenetic relationships with other species were analysed. Finally, demographic analysis using Pairwise Sequentially Markovian Coalescent (PSMC) revealed significant phases in the population history of these Lamprotula species. In summary, our findings provide critical genomic resources that are essential for the conservation of Lamprotula species in China and significantly contribute to our knowledge of the evolutionary history within the family *Unionidae*.

## 2. Materials and methods

### 2.1. Sample collection and DNA extraction

Eight species of *Lamprotula* were included in this study, specifically *L. rochechouarti*, *L. tortuosa*, *L. leai*, *L. caveata*, *L. scripta*, *L. zonata*, *L. fibrosa*, and *L. polysticta*, which were supplied by Wuhu Yangtze River Mollusk Conservation Base of the Ministry of Agriculture and Rural Affairs (Table 1). The specimens included *L. rochechouarti*, *L. scripta*, *L. zonata*, and *L. fibrosa* from Poyang Lake, Jiangxi Province; *L. leai* and *L. caveata* from Jingshan River, Anhui Province; *L. tortuosa* from Leishui River, Hunan Province; and *L. polysticta* from Xihe River, Guangxi Zhuang Autonomous Region. Among these, *L. caveata* had the lowest individual weight at 54.7 g, while *L. polysticta* had the highest at 297.66 g. Detailed measurements of their shell length, shell width, and shell height were provided in Table 1.

**Table 1.** Sample information for the 8 *Lamprotula* species used in this study.

Species	Collection date	Shell length (mm)	Shell width (mm)	Shell height (mm)	Weight (g)	Sample source
*Lamprotula rochechouarti*	2024-4-11	77.44	29.77	57.71	108.4	Poyang Lake, Jiangxi Province, China
*Lamprotula tortuosa*	2024-4-11	106.16	50.6	52.54	264.74	Leishui River, Hengyang, Hunan Province, China
*Lamprotula leai*	2024-4-11	106.83	35.16	55.39	175.93	Jing Shan River, Wuhu, Anhui Province, China
*Lamprotula caveata*	2024-4-11	65.45	26.98	34.27	54.7	Jing Shan River, Wuhu, Anhui Province, China
*Lamprotula scripta*	2024-4-11	86.08	45.71	60.82	202.33	Poyang Lake, Jiangxi Province, China
*Lamprotula zonata*	2024-4-11	85.96	42.46	59.84	180.47	Poyang Lake, Jiangxi Province, China
*Lamprotula fibrosa*	2024-4-11	96.24	42.57	43.23	226.37	Poyang Lake, Jiangxi Province, China
*Lamprotula polysticta*	2024-4-11	97.08	52.73	40.85	297.66	Xihe River, Guilin, Guangxi Zhuang Autonomous Region, China

For genome sequencing, an individual was selected based on its morphological characteristics, and muscle tissue samples were preserved in 95% alcohol to prepare for DNA extraction. The DNA extraction process followed the traditional phenol/chloroform method with RNase A treatment for DNA template purification. Initially, adductor muscle tissues were ground into a fine powder under liquid nitrogen conditions. Subsequently, SDS buffer and proteinase K were added to lyse cells and eliminate contaminants. DNA extraction was performed using a mixture of phenol:chloroform:isoamyl alcohol (25:24:1), and DNA was precipitated by isopropanol. The resulting DNA pellet was washed with chilled 70% ethanol. After removing residual ethanol through evaporation, the DNA was resuspended in sterile water.

### 2.2. Whole-genome sequencing and quality control

Genomic DNA extracted from each *Lamprotula* species was utilized to construct sequencing libraries with fragment lengths ranging from 300 to 400 base pairs (bp). Sequencing was conducted on MGI’s DNBseq-T7 platform using paired-end reads of 2 × 150 bp, in accordance with the manufacturer’s protocols. The raw sequencing data were processed using Fastp (v0.23.2) for quality control.^33^ This involved adapter trimming, filtering out low-quality reads (reads where more than 40% of bases had a quality score below Q15 or contained over 5 ambiguous ‘N’ bases), and removing duplicate reads. Following these steps, clean data for each species were obtained (Table 2).

**Table 2.** High-throughput sequencing data statistics for the 8 *Lamprotula* species.

Species	Library Size (bp)	Sequencing type	Raw data (Gb)	Total clean reads	Total clean bases (Gb)	Q20 (%)	Q30 (%)	GC content (%)
*Lamprotula rochechouarti*	300–400	Paired-end	195.57	1,249,280,958	186.63	98.97	97.45	37.03
*Lamprotula tortuosa*	300–400	Paired-end	248.26	1,558,836,150	232.79	98.88	97.25	35.77
*Lamprotula leai*	300–400	Paired-end	194.70	1,242,405,262	185.73	98.91	97.25	36.08
*Lamprotula caveata*	300–400	Paired-end	232.91	1,476,561,200	220.53	98.85	97.13	36.09
*Lamprotula scripta*	300–400	Paired-end	251.78	1,585,295,636	236.57	98.85	97.18	35.73
*Lamprotula zonata*	300–400	Paired-end	217.41	1,379,786,284	206.00	98.88	97.25	35.62
*Lamprotula fibrosa*	300–400	Paired-end	222.36	1,405,719,326	208.77	99.24	97.80	35.78
*Lamprotula polysticta*	300–400	Paired-end	253.43	1,591,112,558	237.51	99.21	97.60	35.75

To assess potential contamination in the clean data, a random selection of 10,000 paired-end clean reads per species was subjected to BLAST analysis against the NCBI Nucleotide (NT) database (Blastn v2.11.0).^34^ Species with an identity score exceeding 80% were recorded. The number of reads aligning to any species in the NT database, as well as those specifically aligning to the corresponding genus, were counted and their proportions calculated to determine the extent of contamination and to filter out the contaminated reads accordingly. Ultimately, 8 high-quality, contamination-free datasets were obtained, suitable for downstream analyses.

### 2.3. K-mer analysis and ploidy determination of 8 *Lamprotula* species

To investigate the genomic characteristics of 8 *Lamprotula* species, K-mer analysis was conducted to estimate genome size, heterozygosity, and repeat content. The K-mer size for all 8 species was set to 17, and the 17-mers depth distribution was calculated using Jellyfish (v2.2.4).^35^ The 17-mers frequency distribution was found to be consistent with a Poisson distribution, and the peak depth value was determined, representing the average and variance of the associated Poisson distribution. The genome size of each species was estimated using the following equation: G = K-mer-num/K-mer-depth, where K-mer-num was the total number of 17-mers, K-mer-depth was the K-mer depth, and G represents the estimated genome size. This process was facilitated using GCE (v1.0.0) software.^27^ Furthermore, 17-mers with a depth of 1 were considered to be errors, and the error rate was calculated to revise the estimated genome size. During this analysis, the heterozygosity ratio and proportions of repeat sequences for the 8 species were also obtained. To further explore the ploidy of these 8 species, Smudgeplot (v0.2.3.dev) was applied to analyse their genome structures, with parameters set to -k 17 -m100 -ci 1 -cs 10000.^36^

### 2.4. Genome assembly and mitochondrial genome characterization

To assembly the draft genomes of the 8 species, SOAPdenovo (v2.04) was used for genome assembly with the parameters set as: -d 1 -R -K 127 -p 60.^28^ During the assembly process, clean reads were first assembled into contigs. Subsequently, all clean reads were realigned to these contigs, and scaffolds were constructed step by step using paired-end reads with fixed insert sizes, resulting in a scaffold-level genome assembly. Assembly metrics, including N50, N90, and the length of the longest sequence, were obtained using the Seqkit (v2.8.2) tool.^37^

Meanwhile, to obtain their mitochondrial genomes, this study utilized NOVOPlasty (v4.3.1) with the following parameters: Genome Range = 13,000 to 19,500, K-mer = 33, Read Length = 150, Insert size = 300, Single/Paired = PE.^29^ The raw data (with only adapters removed) from the 8 species were assembled. The assembled mitochondrial genomes were manually verified to ensure they formed complete circular sequences.

To assess the consistency of the mitochondrial genome sequences, the cleaned data were aligned to the mitochondrial genomes using bwa (v0.7.12-r1039),^38^ facilitating the calculation of alignment rates and coverage at different depths. Annotation of mitochondrial genomes was performed using mitoZ (v3.6) with the parameters set as mitoz annotate --clade Mollusca --genetic_code 5.^39^ Finally, visualization of the mitochondrial genomes was achieved using both mitoZ (v3.6)^39^ and OGDRAW (v1.3.1).^40^

### 2.5. Identification of SSRs

To identify the types and numbers of SSRs in the 8 *Lamprotula* species, the Perl script ‘misa.pl’ from the MISA (v2.1) software was employed to analyse potential microsatellite motifs within their genomes.^41^ The search parameters were configured to detect di-, tri-, tetra-, penta-, and hexa-nucleotide repeats with minimum repeat lengths of 6, 5, 5, 5, and 5 repeats, respectively. Following the identification of SSRs, their distribution characteristics were visualized using the ggplot2 package in R,^42^ providing a comprehensive overview of SSR patterns across the species.

### 2.7 Construction of phylogenetic tree based on 8 mitochondrial genomes

Mitochondrial genomes (mitogenomes) play a crucial role in evolutionary studies.^43^ Consequently, the utilization of complete mitogenomes for phylogenetic investigations has become standard practice.^39^ To elucidate the evolutionary relationships among *Lamprotula* species, this study employed a strategy based on their entire mitogenome sequences. Mitochondrial genome sequences were aligned using MAFFT (v7.526) with the L-INS-i algorithm (--localpair), implementing 1,000 iterative refinements for maximum accuracy (--maxiterate 1000).^44^ Phylogenetic inference was performed under the generalized time-reversible nucleotide substitution model using FastTree (v2.1.11).^45^ Node support was assessed with 1,000 bootstrap replicates (-boot 1000), and branch lengths were optimized with maximum-likelihood approximation.

### 2.8 Inference of population size dynamics for 8 *Lamprotula* species

To infer the population size history of these *Lamprotula* species, the PSMC method (v0.6.5) was utilized.^32^ First, clean reads were aligned to the assembled genome using BWA-MEM2,^38^ generating BAM files. Following this, the ‘fq2psmcfa’ and ‘splitfa’ tools from the PSMC software suite were employed to prepare the input files necessary for PSMC modelling.

The PSMC analysis was conducted with specific parameters: -N25 to define the number of algorithm cycles and -t15 to establish the upper limit for the most recent common ancestor. The resulting population history was visualized using the ‘psmc_plot.pl’ script, applying a substitution rate of 1.16e-8 and assuming a generation time of 2 years.

## 3. Results

### 3.1. High-throughput sequencing and K-mer analysis of 8 *Lamprotula* species genomes

This study included 8 *Lamprotula* species collected in 2024 from 4 provinces in China, with their weights ranging from 54.7 to 297.66 g, for high-throughput paired-end sequencing (Table 1). A library with a size range of 300 to 400 base pairs was constructed for each species and sequenced using DNBSeq technology. The raw data obtained for *L. rochechouarti*, *L. tortuosa*, *L. leai*, *L. caveata*, *L. scripta*, *L. zonata*, *L. fibrosa*, and *L. polysticta* were 195.57, 248.26, 194.70, 232.91, 251.78, 217.41, 222.36, and 253.43 Gb, respectively (Table 2). Subsequently, the data were quality-controlled using fastp, resulting in clean data with Q30 scores greater than 97% and Q20 scores greater than 98% for all 8 species. Among these, the GC content in the clean data of *L. zonata* was the lowest at 35.62%, while that of *L. rochechouarti* was the highest at 37.03%. Additionally, the NT database comparison results indicated that the top 3 species identified through BLAST searches were predominantly members of the *Family Unionidae* (Table S1). This finding confirmed that there was no significant exogenous contamination during the library construction process. Ultimately, the clean data sizes for *L. rochechouarti*, *L. tortuosa*, *L. leai*, *L. caveata*, *L. scripta*, *L. zonata*, *L. fibrosa*, and *L. polysticta* were 186.63, 232.79, 185.73, 220.53, 236.57, 206.00, 208.77, and 237.51 Gb, respectively (Table 2).

**Fig. 1.**
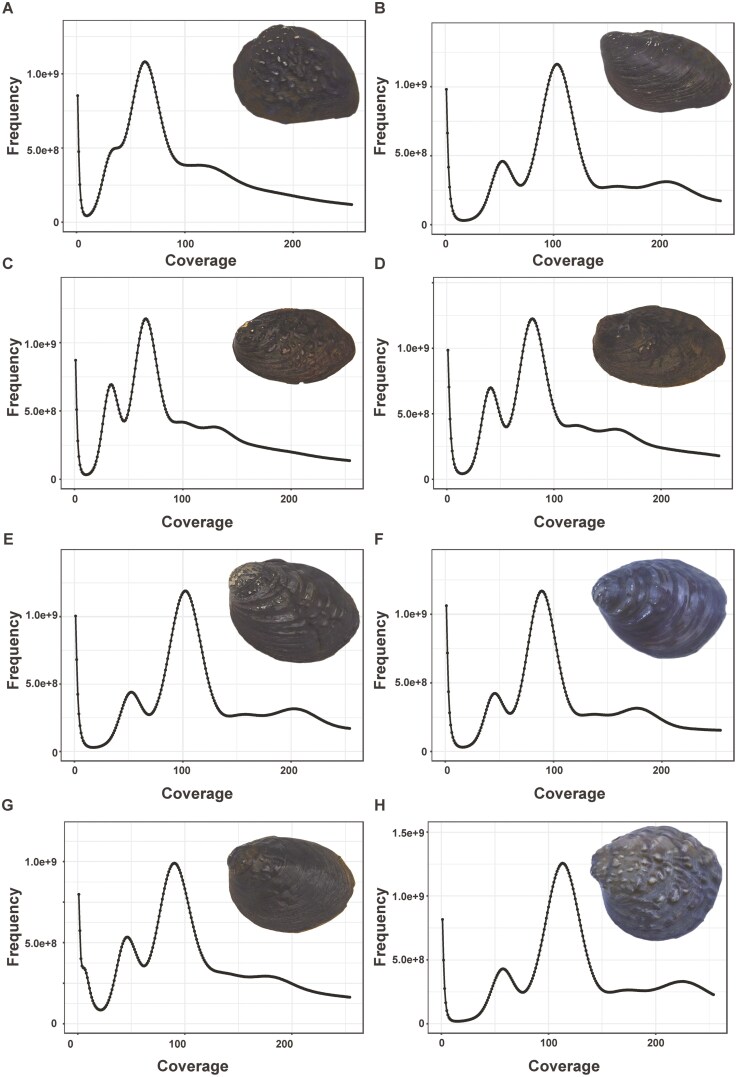
K-mer (K = 17) analysis for genomic characterization of 8 *Lamprotula* freshwater bivalve species. a–h) *L. rochechouarti*, *L. tortuosa*, *L. leai*, *L. caveata*, *L. scripta*, *L. zonata*, *L. fibrosa*, and *L. polysticta*, respectively. The *x* axis indicates K-mer depth, and the *y* axis indicates K-mer frequency.

**Fig. 2.**
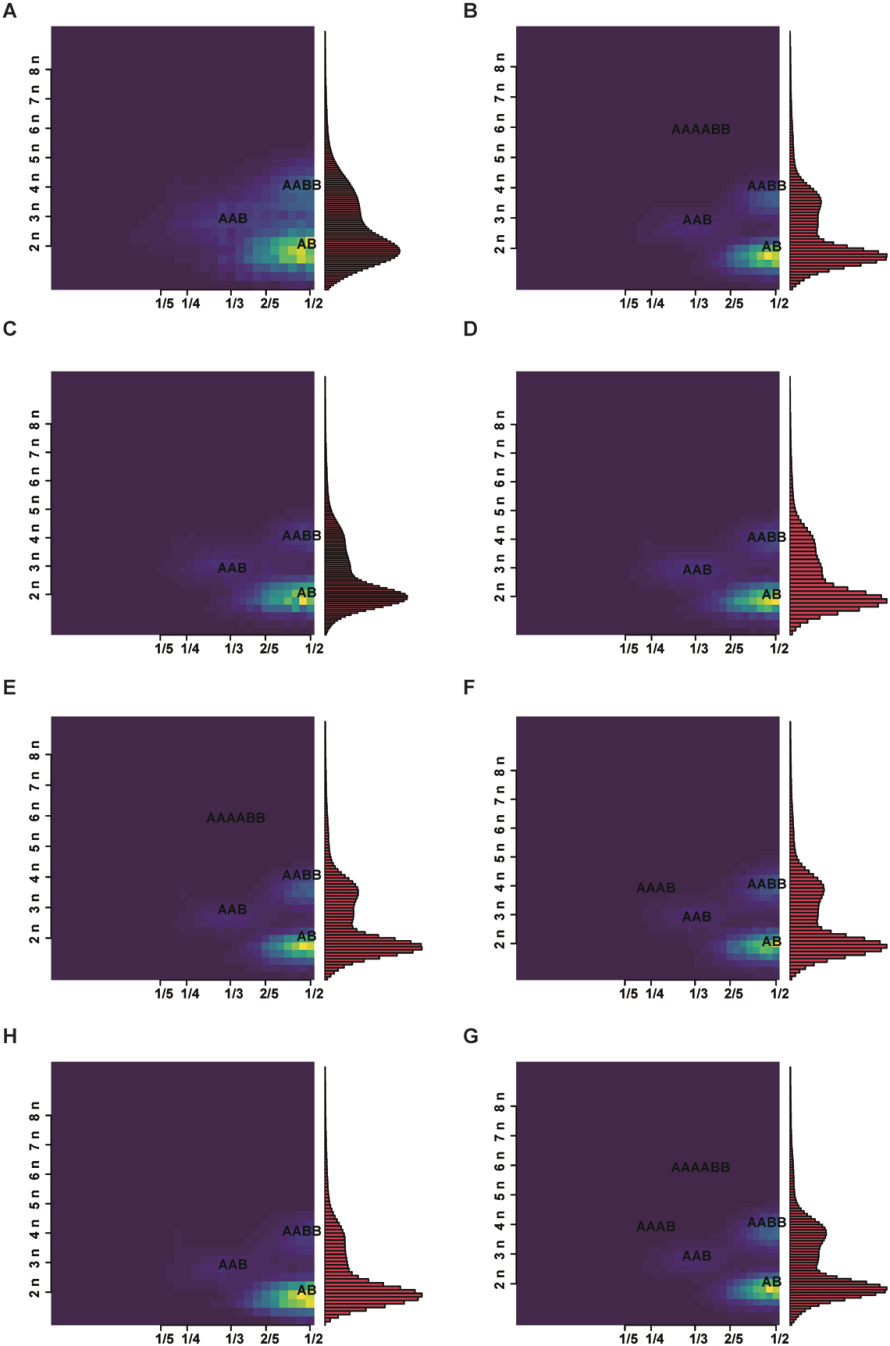
Smudgeplot estimation of ploidy for 8 *Lamprotula* freshwater bivalve species. a–h) *L. rochechouarti*, *L. tortuosa*, *L. leai*, *L. caveata*, L. scripta, *L. zonata*, *L. fibrosa*, and *L. polysticta*, respectively. The *x* axis represents relative coverage (CovB/(CovA + CovB)), the *y* axis represents total coverage (CovA + CovB), and colour indicates k-mer pair frequency.

K-mer analysis was employed to estimate the genome size, heterozygosity ratio, and repeat content of 8 *Lamprotula* species genomes, with a uniform K-value of 17 set for all analyses (Table 3). The expected K-mer depth was calculated as 60, 101, 64, 77, 100, 87, 87, and 111 for the respective *Lamprotula* species (Fig. 1). Using the formula genome size = (total number of k-mers)/(peak depth), the initial estimated genome sizes for *L. rochechouarti*, *L. tortuosa*, *L. leai*, *L. caveata*, *L. scripta*, *L. zonata*, *L. fibrosa*, and *L. polysticta* were determined to be 2.66, 2.03, 2.59, 2.56, 2.07, 2.05, 2.14, and 1.89 Gb, respectively. After correction using Genome Character Estimator, the final estimated genome sizes ranged from 1.89 to 2.65 Gb for these species. All 8 species exhibited high heterozygosity rates (greater than 0.8) and high repeat content (exceeding 60%), posing significant challenges for generating high-quality reference genomes in subsequent studies. According to the Smudgeplot analysis results, the proportions of AB-type k-mers for the 8 species were 0.61, 0.56, 0.62, 0.60, 0.54, 0.56, 0.66, and 0.52 (Fig. 2 and Table S2). When combined with the previous findings, this suggested that the genomes of these 8 species were all large, highly heterozygous, and highly repetitive, typical of complex diploid genomes.

**Table 3.** Results of K-mer analysis for 8 *Lamprotula* species.

Species	K-mer size	Total K-mer	K-mer depth	Esitimated genome size (bp)	Revised genome size (bp)	Heterozygous ratio (%)	Repeat (%)
*Lamprotula rochechouarti*	17	166,567,551,905	60	2,662,230,000	2,648,606,184	0.99	65.78
*Lamprotula tortuosa*	17	207,744,257,664	101	2,028,720,000	2,019,138,943	0.87	63.47
*Lamprotula leai*	17	165,777,903,081	64	2,590,279,736	2,576,668,228	1.42	63.46
*Lamprotula caveata*	17	196,808,135,862	77	2,555,949,816	2,543,155,844	1.35	62.70
*Lamprotula scripta*	17	211,106,855,124	100	2,067,440,000	2,057,609,126	0.84	64.52
*Lamprotula zonata*	17	183,837,950,251	87	2,052,000,000	2,040,140,119	0.86	64.35
*Lamprotula fibrosa*	17	186,246,494,094	87	2,140,764,300	2,131,595,254	1.63	62.25
*Lamprotula polysticta*	17	212,018,477,813	111	1,894,800,000	1,887,507,685	0.82	61.67

### 3.2. Genome assembly and mitochondrial genome assembly of 8 *Lamprotula* species

To explore further genomic characteristics of these 8 *Lamprotula* species, SOAPDenovo2 was utilized for assembling their draft genomes. The results showed that *L. caveata* had both the largest genome size and the longest sequence among these 8 species. Conversely, *L. polysticta* had the smallest genome size at the contig level, measuring 2.26 Gb compared to *L. caveata*’s 3.33 Gb. At the scaffold level, the sizes ranged from 2.35 Gb to 3.47 Gb (Table 4). Despite having the smallest draft genome size, *L. polysticta* exhibited the largest scaffold N50 of 2.28 kb, while *L. fibrosa* had shown the smallest at 0.95 kb. Compared to the estimated sizes from K-mer analysis, the assembled genome sizes of these 8 species were on average 22.58% larger than expected, a discrepancy that was closely related to the high heterozygosity characteristic of their genomes. The GC content across the draft genomes of these 8 species had been largely consistent, fluctuating between 35.08% and 35.82%, with an average of 35.29%. Overall, this information had provided a solid foundation for constructing high-quality reference genomes in future studies.

**Table 4.** Statistics of genome assembly for 8 *Lamprotula* species.

Species	Total contig length (bp)	Total scaffolds	Total scaffold length (bp)	Scaffold N50 (bp)	Largest scaffold (bp)	GC (%)
*Lamprotula rochechouarti*	3,065,532,295	4,038,592	3,209,067,863	1,681	85,765	35.82
*Lamprotula tortuosa*	2,325,850,842	2,911,977	2,411,778,601	1,730	88,999	35.14
*Lamprotula leai*	2,926,540,197	3,281,778	3,061,408,379	1,687	66,635	35.50
*Lamprotula caveata*	3,327,384,479	4,036,935	3,465,891,362	2,008	105,433	35.48
*Lamprotula scripta*	2,322,900,503	3,098,426	2,424,923,496	1,754	99,338	35.08
*Lamprotula zonata*	2,283,197,578	2,902,651	2,382,349,442	1,787	79,547	35.10
*Lamprotula fibrosa*	2,568,870,412	4,567,553	2,685,642,119	946	48,928	35.12
*Lamprotula polysticta*	2,261,352,812	2,520,167	2,347,051,140	2,278	89,097	35.09

Mitochondria played multiple crucial roles in mussel species, significantly contributing to the maintenance of normal physiological functions and adaptation to environmental changes.^43,46^ To obtain complete mitochondrial genome sequences, high-quality sequencing data were generated, with clean data sizes ranging from 9.70 Gb to 12.60 Gb, and Q20 values reaching approximately 99% (Table S3). The assembly results indicated that all 8 species obtained high-quality circular mitochondrial genomes, with sizes ranging from 15.69 kb to 17.13 kb, and GC contents fluctuating between 36.36% and 40.77% (Table 5).

**Table 5.** Assembly and annotation of mitochondrial genomes for 8 *Lamprotula* species.

	*Lamprotula rochechouarti*	*Lamprotula tortuosa*	*Lamprotula leai*	*Lamprotula caveata*	*Lamprotula scripta*	*Lamprotula zonata*	*Lamprotula fibrosa*	*Lamprotula polysticta*
Genome length (bp)	16,111	15,724	16,118	17,132	15,689	15,699	15,700	15,693
Contig Num	1	1	1	1	1	1	1	1
GC content (%)	39.07	36.36	39.83	40.77	37.1	37.01	36.62	36.65
Circularized	Yes	Yes	Yes	Yes	Yes	Yes	Yes	Yes
Number of protein-coding genes	13	13	13	13	13	13	13	13
Number of tRNA genes	22	22	22	22	22	22	22	22
Number of rRNA genes	2	2	2	2	2	2	2	2
Total genes	37	37	37	37	37	37	37	37

**Fig. 3.**
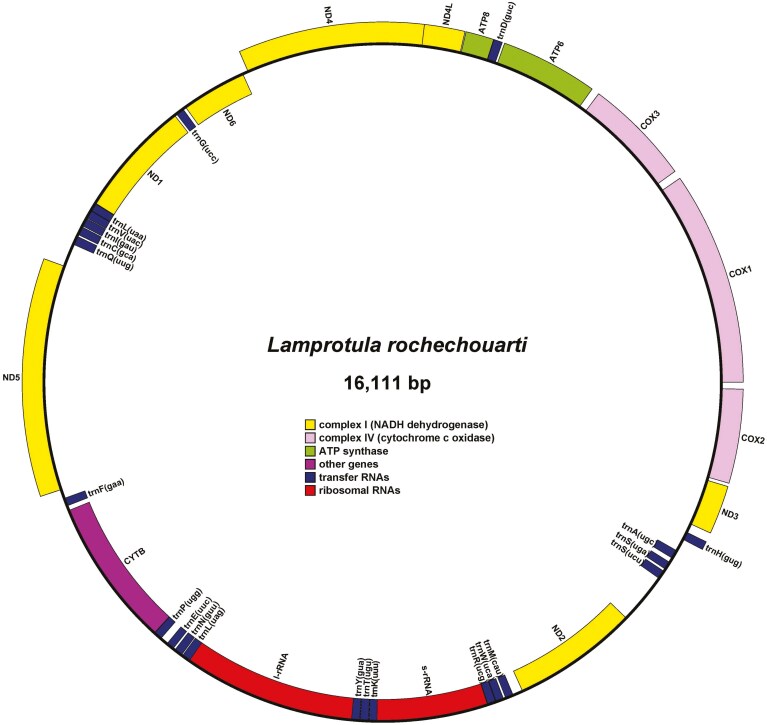
Mitochondrial genome structure of *L. rochechouarti*. Yellow blocks represent NADH dehydrogenase, pink blocks represent cytochrome c oxidase, green blocks represent ATP synthase, blue blocks represent tRNA, red blocks represent rRNA, and purple-red blocks represent other genes.

To assess the sequence consistency of these mitochondrial genomes, the quality-controlled data were aligned to the mitochondrial genomes using bwa.^38^ The results showed an average read mapping rate of 96.54%, with the mitochondrial genomes achieving 100% average coverage. Regions with coverage greater than 20x accounted for an average of 98.98%, demonstrating excellent sequence consistency among them (Fig. S1 and Table S4). Additionally, after annotating the protein-coding genes in their mitochondrial genomes, it was found that all 8 species contained 37 genes, including 13 protein-coding genes, 22 tRNAs, and 2 rRNAs (Table 5). Visualization of their mitochondrial genomes revealed that the gene structures and positions were highly conserved (Fig. 3 and Figs. S2–S8).

### 3.3. Characterization of potential microsatellite markers

**Fig. 4.**
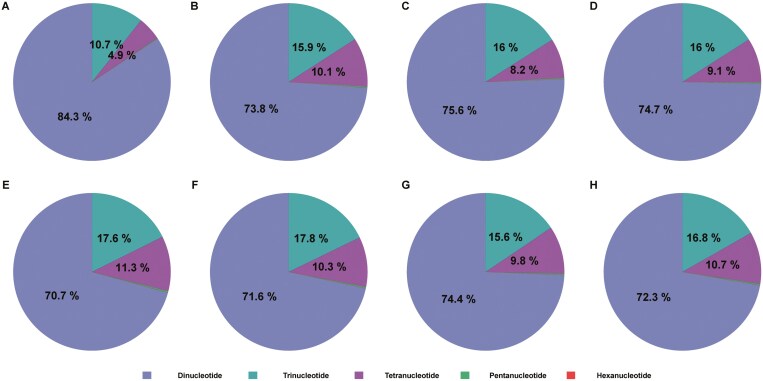
The distribution of different repeat motif types within 8 *Lamprotula* freshwater bivalve species. a–h) *L. rochechouarti*, *L. tortuosa*, *L. leai*, *L. caveata*, *L. scripta*, *L. zonata*, *L. fibrosa*, and *L. polysticta*, respectively.

Based on the draft genomes of 8 *Lamprotula* species, a total of 1,282,789, 826,136, 983,887, 1,263,379, 964,217, 845,103, 892,958, and 901,611 SSRs were identified, respectively (Table 6). Among the microsatellite motif types observed in these species, dinucleotide repeats were predominant (ranging from 70.73% to 84.29%), followed by trinucleotide repeats (10.72% to 17.80%), tetranucleotide repeats (4.85% to 11.30%), pentanucleotide repeats (0.12% to 0.30%), and hexa-nucleotide repeats (0.01% to 0.02%) (Fig. 4 and Table S5). The proportion of microsatellite motifs decreased as the repeat unit length increased. The SSR density across these species varied from 321 per Mb in *L. leai* to 400 per Mb in *L. rochechouarti* (Table 6). These findings facilitated the development of molecular markers for species identification and contributed further to the population genetics studies of *Lamprotula* species.^47,48^

**Table 6.** Microsatellite motif statistics of 8 *Lamprotula* species.

	*Lamprotula rochechouarti*	*Lamprotula tortuosa*	*Lamprotula leai*	*Lamprotula caveata*	*Lamprotula scripta*	*Lamprotula zonata*	*Lamprotula fibrosa*	*Lamprotula polysticta*
Total size of examined sequences (bp)	3,209,067,863	2,411,778,601	3,061,408,379	3,465,891,362	2,424,923,496	2,382,349,442	2,685,642,119	2,347,051,140
Total number of SSRs	1,282,789	826,136	983,887	1,263,379	964,217	845,103	892,958	901,611
SSR density (SSR number/genome size)	399.74	342.54	321.38	364.52	397.63	354.74	332.49	384.15
Number of dinucleotide repeats	1,081,367	609,589	743,565	943,620	682,004	605,425	664,033	651,442
Number of trinucleotide repeats	137,560	131,147	157,416	201,592	170,159	150,428	139,029	151,305
Number of tetranucleotide repeats	62,200	83,220	80,796	115,182	108,965	86,920	87,574	96,299
Number of pentanucleotide repeats	1,531	2,012	1,939	2,716	2,859	2,167	2,150	2,388
Number of hexa-nucleotide repeats	131	168	171	269	230	163	172	177

### 3.4. Phylogenetic relationship of 8 *Lamprotula* species based on complete mitochondrial genome sequences

**Fig. 5.**
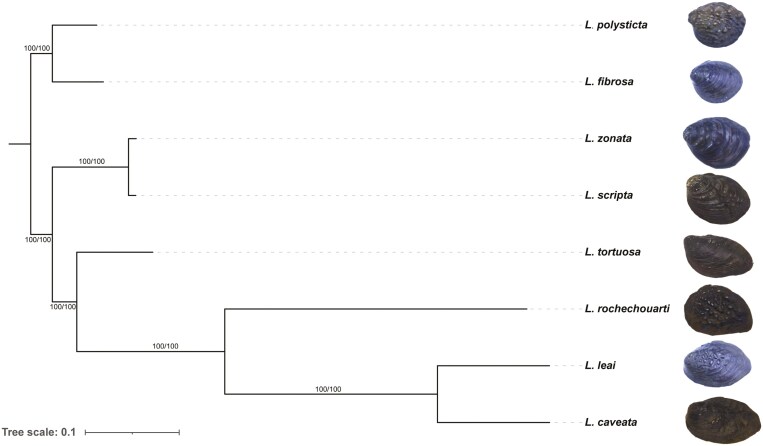
The phylogenetic tree was inferred from the complete mitochondrial genomes of 8 *Lamprotula* freshwater bivalve species.

To resolve the evolutionary relationships within the genus *Lamprotula*, a phylogenetic tree was constructed based on complete mitochondrial genome sequences. The resulting maximum-likelihood phylogenetic tree revealed 2 major clades within *Lamprotula* (Fig. 5). One clade included *L. polysticta* and *L. fibrosa*, indicating close genetic relationships of these 2 species. The second major clade comprised *L. zonata*, *L. scripta*, *L. tortuosa*, *L. rochechouarti*, *L. leai*, and *L. caveata*, with *L. leai* and *L. caveata* forming a well-supported sister group. All nodes were strongly supported with bootstrap values of 100, underscoring the robustness of the phylogenetic relationships inferred. Representative shell morphologies of each species are shown alongside the tree, illustrating morphological variation in correspondence with phylogenetic divergence.

### 3.5. The population size dynamics of 8 *Lamprotula* species

**Fig. 6.**
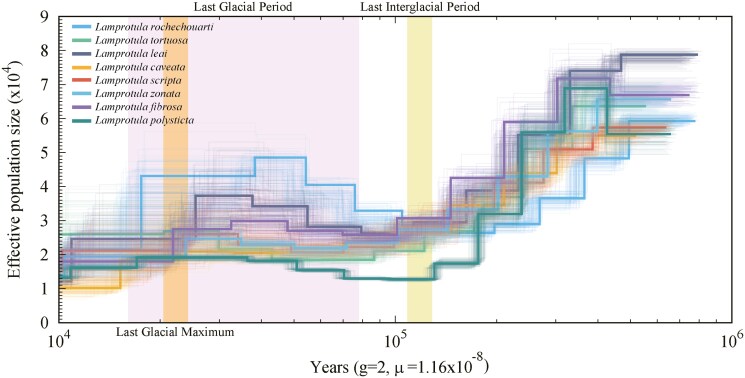
Effective population size estimates using PSMC for 8 *Lamprotula* freshwater bivalve species. The *x* axis represents time before present (Kya), ranging from 10 to 1,000 thousand years (left to right). The *y* axis represents Ne. Parameter ‘g’ denotes generation time (years), and ‘μ’ represents mutation rate.

PSMC analysis revealed that the 8 *Lamprotula* species experienced population bottlenecks during the past million years (Fig. 6). During the Last Interglacial Period (~130 to 116 Kya), the effective population size of all 8 *Lamprotula* species decreased at a constant rate from its peak. As global temperatures rose, sea levels increased, and glaciers melted during this period, the sustained decline in population size may be associated with habitat changes, such as the loss of shallow coastal areas and alterations in ecosystem structure, making it difficult for the species to maintain large populations.^49,50^ During the Last Glacial Period (~70 to 15 Kya), the effective population sizes of these 8 species began to increase at a relatively stable rate. Subsequently, their population sizes showed a significant decline again during the Last Glacial Maximum, reaching their lowest points. Among the 8 *Lamprotula* species, *L. tortuosa* exhibited notably larger effective population sizes compared to the other 7 species. Throughout the Pleistocene Glacial Epoch, *L. rochechouarti*’s population size fluctuated more significantly than the other species.

## 4. Discussion

This study represented the first comprehensive genome sequencing analysis of 8 *Lamprotula* species. Utilizing high-depth clean data, K-mer analysis had confirmed that these species possessed complex diploid genomes characterized by high heterozygosity and high proportions of repetitive sequences. Concurrently, the mitochondrial genome sizes of these 8 species had shown modest variation, ranging from 15.69 kb to 17.13 kb, with GC content fluctuating between 36.36% and 40.77%. Upon annotating the protein-coding genes within their mitochondrial genomes, it was found that all 8 species contained a total of 37 genes, comprising 13 protein-coding genes, 22 tRNAs, and 2 rRNAs. Among the observed microsatellite motif types in these species, dinucleotide repeats had been predominant, accounting for 70.73% to 84.29% of the motifs. In the phylogenetic tree constructed, *L. leai* and *L. caveata* had diverged early from the other 6 *Lamprotula* species, forming a distinct clade indicative of greater evolutionary divergence, likely influenced by their unique environmental conditions and characteristics.^13,51^ Within the clade containing the remaining 6 species, *L. scripta* and *L. zonata* had exhibited closer genetic relationships. Furthermore, PSMC analysis had revealed that these 8 *Lamprotula* species had experienced population bottlenecks over the past million years.

Considering the genomic complexity of these 8 species, the short-read sequencing strategy employed in this study presents inherent limitations. Firstly, substantial discrepancies exist between the draft assemblies and k-mer-based size estimates. Specifically, k-mer analysis predicted genome sizes of 1.89-2.65 Gb, approximately 22% smaller on average than the SOAPdenovo2 assemblies (2.35 to 3.47 Gb). This pattern aligns with observations in other aquatic genomes and stems from fundamental challenges in resolving repetitive and heterozygous regions with short reads.^52–54^ High genomic repetitiveness and heterozygosity frequently cause inflated assembly sizes and fragmentation. When short-read assemblies fail to resolve these complex regions correctly, these sequences may be artificially duplicated, increasing assembly size, or cause contig fragmentation. Second, fragmented assemblies affect downstream analyses. For SSR marker identification, longer SSR motifs spanning fragment boundaries become difficult to identify. For PSMC inference, fragmentation introduces biases in heterozygous SNP detection through haplotype breakpoints where short contigs disrupt linkage disequilibrium blocks, impairing phasing accuracy. Boundary artefacts causing elevated spurious heterozygosity near contig ends and coverage bias from uneven mapping in repetitive regions that inflates false heterozygosity. These artefacts consequently distort PSMC-inferred population size events, promote overestimation of recent effective population size and reduce resolution of recent demographic events, particularly for recent bottlenecks. Future work could leverage the integration of short-read sequencing, long-read sequencing, and Hi-C mapping technologies to produce a high-quality, chromosome-scale reference genome for these freshwater mussels.^19,55,56^ Combined with comprehensive whole-genome annotation and comparative genomics analyses, this will play a crucial role in deepening our understanding of the evolutionary mechanisms and environmental adaptations of these species. Moreover, it will furnish materials for analysing the endangered mechanisms of *Lamprotula* populations. Additionally, this will also offer crucial genetic resources for enhancing their significant traits, conducting selective breeding, and facilitating aquaculture practices.

Furthermore, while this study revealed the demographic dynamics of *Lamprotula* species using PSMC analyses based on single samples from 8 species, these inferred population size changes require validation with larger datasets. Crucially, cryptic diversity or significant population structure might exist within *Lamprotula* species, complexities that a single sample cannot capture.^13^ For instance, if the sampled individual belonged to a genetically distinct subpopulation, the PSMC-inferred effective population size might only reflect the history of that subpopulation, not the entire species.^57^ Moreover, PSMC assumes a randomly mating population; the presence of cryptic diversity or population structure could violate this assumption, potentially leading to misinterpretation of bottleneck or expansion events.^58^ Future studies should validate these findings and enhance understanding by incorporating broader sampling per species and integrating complementary population genetics methods to provide a more comprehensive picture of *Lamprotula*’s evolutionary history.

Mitochondria play a crucial role in the biological functions of freshwater mussels, involving multiple aspects such as energy metabolism, cell apoptosis, genetic diversity, and evolutionary studies.^43,46^ Therefore, characterizing the mitochondrial genome of *Lamprotula* was essential. In this study, NOVOPlasty was employed for the *de novo* assembly of their mitochondrial genomes,^29^ a tool that has proven to be highly effective for this purpose. The mitochondrial genomes of these 8 species were found to be similar in size and circular in structure, with high sequence identity, confirming the reliability of these mitochondrial genomes. When compared with publicly available mitochondrial data from NCBI, the mitochondrial genome size of *L. tortuosa* assembled in this study is 15,724 bp, while the previously reported sequence (NC_021404.1) was 15,722 bp.^59^ Their GC contents are 36.36% and 36.25%, respectively. The consistency not only validated the accuracy of the assembly process but also underscored the utility of NOVOPlasty in generating reliable mitochondrial genome. These mitochondrial datasets provided significant contributions to the genetic resources of *Lamprotula*, enhancing our understanding of their genetic makeup and evolutionary history.

Overall, the natural resources of the *Lamprotula* species had drastically diminished, pushing global populations to the brink of depletion.^8,10,16,60^ The urgency to adopt effective measures for the protection and proliferation of these species could not have been overstated. Our research provided genomic data resources for 8 *Lamprotula* species, which held significant implications for the ecology and conservation of *Lamprotula* species. Additionally, our findings contributed to uncovering critical genetic traits essential for artificial breeding and seedling cultivation of *Lamprotula*, thus facilitating initiatives focussed on the preservation and enhancement of *Lamprotula* resources.

## Supplementary Material

dsaf020_suppl_Supplementary_Table_1

dsaf020_suppl_Supplementary_Figure_1

## Data Availability

Illumina paired-end reads have been deposited in NCBI/BioProject PRJNA1301049. The whole-genome sequence data reported in this paper have been deposited in NCBI, under accession numbers (SAMN50434169, SAMN50434170, SAMN50434171, SAMN50434172, SAMN50434173, SAMN50434174, SAMN50434175, SAMN50434176). The mitochondrial genomes of these 8 species are available in Figshare (https://doi.org/10.6084/m9.figshare.29498255).
