# Supplementary material for: Genome survey and evolutionary analysis of 8 Lamprotula species: SSR profiling, mitochondrial characterization, and population dynamics inference
Source: DNA Res. 2025 Aug 9;32(5):dsaf020. doi: 10.1093/dnares/dsaf020 (PMC12454935; doi:10.1093/dnares/dsaf020)
Supplement: dsaf020_suppl_Supplementary_Table_1 [file dsaf020_suppl_supplementary_table_1.docx]

| **Supplementary Table 1. Results of NT contamination assessment for eight *Lamprotula* species.** | | | | | |
| --- | --- | --- | --- | --- | --- |
| **Species** | **Hit Number** | **Genus** | **Blast Number** | **Percentage of hits (%)** | **Median identity (%)** |
| *Lamprotula rochechouarti* | 542 | *Hyriopsis* | 150 | 27.68 | 97.33 |
|  | 542 | *Cristaria* | 40 | 7.38 | 87.18 |
|  | 542 | *Amblema* | 32 | 5.90 | 94.64 |
| *Lamprotula tortuosa* | 713 | *Hyriopsis* | 245 | 34.36 | 90.00 |
|  | 713 | *Cristaria* | 73 | 10.24 | 92.00 |
|  | 713 | *Lamprotula* | 50 | 7.01 | 98.67 |
| *Lamprotula leai* | 862 | *Hyriopsis* | 247 | 28.65 | 91.34 |
|  | 862 | *Lamprotula* | 85 | 9.86 | 93.33 |
|  | 862 | *Mastacembelus* | 56 | 6.5 | 95.36 |
| *Lamprotula caveata* | 771 | *Hyriopsis* | 230 | 29.83 | 91.12 |
|  | 771 | *Lamprotula* | 79 | 10.25 | 92.97 |
|  | 771 | *Cristaria* | 53 | 6.97 | 92 |
| *Lamprotula scripta* | 736 | *Hyriopsis* | 250 | 33.97 | 89.66 |
|  | 736 | *Aculamprotula* | 75 | 10.19 | 100 |
|  | 736 | *Cristaria* | 61 | 8.29 | 92.67 |
| *Lamprotula zonata* | 654 | *Hyriopsis* | 222 | 33.94 | 91.05 |
|  | 654 | *Cristaria* | 54 | 8.26 | 91.93 |
|  | 654 | *Aculamprotula* | 46 | 7.03 | 98.67 |
| *Lamprotula fibrosa* | 769 | *Hyriopsis* | 215 | 27.96 | 90.07 |
|  | 769 | *Tridacna* | 78 | 10.14 | 87.72 |
|  | 769 | *Cristaria* | 55 | 7.15 | 92.52 |
| *Lamprotula polysticta* | 758 | *Hyriopsis* | 245 | 32.32 | 89.81 |
|  | 758 | *Cristaria* | 61 | 8.05 | 91.33 |
|  | 758 | *Tridacna* | 58 | 7.65 | 87.72 |
| ***Hit number**: The number of reads that have alignment results in the NT database. **Genus**: The genus to which the reads are aligned. **Blast number**: The number of reads aligned to this genus. **Percentage of hits (%)**: The proportion of reads aligned to this genus out of the total aligned reads (Blast number / Hit number). **Median identity (%)**: The median percentage of sequence alignment identity. **Note:** The NT assessment results only display information for the top three genera with the highest number of aligned reads for each species. | | | | | |

| **Supplementary Table 2. Results of smudgeplot ploidy analysis for eight *Lamprotula* species.** | | | | |  |
| --- | --- | --- | --- | --- | --- |
| **Species** | **Peak** | **Total kmers** | **Proportion of kmers** | **Summit B/(A + B)** | **Summit A + B** |
| *Lamprotula rochechouarti* | AB | 66,329,159 | 0.61 | 0.49 | 66 |
|  | AABB | 23,676,576 | 0.22 | 0.48 | 117.27 |
|  | AAB | 18,083,452 | 0.17 | 0.32 | 83.09 |
| *Lamprotula tortuosa* | AB | 55,378,872 | 0.56 | 0.48 | 96.58 |
|  | AABB | 22,251,285 | 0.22 | 0.49 | 186.15 |
|  | AAB | 19,226,961 | 0.19 | 0.34 | 147.76 |
|  | AAAABB | 2,398,144 | 0.02 | 0.34 | 301.31 |
| *Lamprotula leai* | AB | 87,392,503 | 0.62 | 0.48 | 56.15 |
|  | AAB | 30,750,054 | 0.22 | 0.34 | 87.39 |
|  | AABB | 22,355,094 | 0.16 | 0.48 | 126.45 |
| *Lamprotula caveata* | AB | 80,625,129 | 0.6 | 0.49 | 72.22 |
|  | AAB | 31,538,080 | 0.23 | 0.34 | 110.64 |
|  | AABB | 22,691,828 | 0.17 | 0.49 | 149.05 |
| *Lamprotula scripta* | AB | 52,421,689 | 0.54 | 0.48 | 96.07 |
|  | AABB | 22,907,490 | 0.24 | 0.49 | 184.84 |
|  | AAB | 19,757,106 | 0.2 | 0.34 | 146.79 |
|  | AAAABB | 2,259,101 | 0.02 | 0.34 | 286.29 |
| *Lamprotula zonata* | AB | 51,830,686 | 0.56 | 0.49 | 86.58 |
|  | AABB | 23,606,640 | 0.26 | 0.49 | 161.6 |
|  | AAB | 14,914,872 | 0.16 | 0.33 | 124.09 |
|  | AAAB | 2,214,271 | 0.02 | 0.26 | 161.6 |
| *Lamprotula fibrosa* | AB | 76,972,396 | 0.66 | 0.48 | 86.69 |
|  | AAB | 22,119,190 | 0.19 | 0.32 | 124.15 |
|  | AABB | 17,780,036 | 0.15 | 0.49 | 161.6 |
| *Lamprotula polysticta* | AB | 49,707,704 | 0.52 | 0.48 | 106.24 |
|  | AABB | 24,002,988 | 0.25 | 0.49 | 204.04 |
|  | AAB | 14,783,529 | 0.15 | 0.33 | 167.36 |
|  | AAAB | 3,978,530 | 0.04 | 0.26 | 204.04 |
|  | AAAABB | 2,991,920 | 0.03 | 0.34 | 314.06 |

| **Supplementary Table 3.Clean data statistics for mitochondrial assembly of eight *Lamprotula* species.** | | | | |
| --- | --- | --- | --- | --- |
| **Species** | **Total clean read (M)** | **Total clean bases** | **Q20(%)** | **Q30(%)** |
| *Lamprotula rochechouarti* | 65.18 | 9.73 | 98.97 | 97.45 |
| *Lamprotula tortuosa* | 82.72 | 12.34 | 98.89 | 97.26 |
| *Lamprotula leai* | 64.89 | 9.7 | 98.91 | 97.25 |
| *Lamprotula caveata* | 77.62 | 11.59 | 98.85 | 97.12 |
| *Lamprotula scripta* | 83.9 | 12.51 | 98.86 | 97.18 |
| *Lamprotula zonata* | 72.46 | 10.81 | 98.89 | 97.25 |
| *Lamprotula fibrosa* | 74.1 | 11 | 99.24 | 97.79 |
| *Lamprotula polysticta* | 84.45 | 12.6 | 99.22 | 97.6 |

| **Supplementary Table 4.Clean reads coverage and depth for mitochondrial genomes of eight *Lamprotula* species.** | | | | | | | |
| --- | --- | --- | --- | --- | --- | --- | --- |
| **Species** | **Mapping rate(%)** | **Paired mapping rate(%)** | **Average sequencing depth** | **Coverage (%)** | **Coverage at least 4X(%)** | **Coverage at least 10X(%)** | **Coverage at least 20X(%)** |
| *Lamprotula rochechouarti* | 98.06 | 95.61 | 177.67 | 100 | 99.96 | 98.66 | 96.88 |
| *Lamprotula tortuosa* | 98.01 | 95.46 | 179.73 | 100 | 100 | 100 | 100 |
| *Lamprotula leai* | 96.25 | 91.97 | 171.96 | 100 | 100 | 99.22 | 96.39 |
| *Lamprotula caveata* | 97.38 | 93.95 | 162.89 | 100 | 100 | 100 | 99.58 |
| *Lamprotula scripta* | 97.62 | 94.51 | 178.99 | 100 | 100 | 100 | 99.96 |
| *Lamprotula zonata* | 97.33 | 94.28 | 179.08 | 100 | 100 | 100 | 99.51 |
| *Lamprotula fibrosa* | 90.32 | 80.2 | 156.81 | 100 | 100 | 100 | 100 |
| *Lamprotula polysticta* | 97.37 | 94.05 | 178.85 | 100 | 100 | 99.96 | 99.51 |

| **Supplementary Table 5.Proportions of different motif types of microsatellites for eight *Lamprotula* species.** | | | | | |
| --- | --- | --- | --- | --- | --- |
| **Species** | **Dinucleotide repeats** | **Trinucleotide repeats** | **Tetranucleotide repeats** | **Pentanucleotide repeats** | **Hexanucleotide repeats** |
| *Lamprotula rochechouarti* | 84.29% | 10.72% | 4.85% | 0.12% | 0.01% |
| *Lamprotula tortuosa* | 73.79% | 15.87% | 10.07% | 0.24% | 0.02% |
| *Lamprotula leai* | 75.57% | 16.00% | 8.21% | 0.20% | 0.02% |
| *Lamprotula caveata* | 74.69% | 15.96% | 9.12% | 0.21% | 0.02% |
| *Lamprotula scripta* | 70.73% | 17.65% | 11.30% | 0.30% | 0.02% |
| *Lamprotula zonata* | 71.64% | 17.80% | 10.29% | 0.26% | 0.02% |
| *Lamprotula fibrosa* | 74.37% | 15.57% | 9.81% | 0.24% | 0.02% |
| *Lamprotula polysticta* | 72.26% | 16.78% | 10.68% | 0.26% | 0.02% |
