# Supplementary material for: Genome survey and evolutionary analysis of 8 Lamprotula species: SSR profiling, mitochondrial characterization, and population dynamics inference
Source: DNA Res. 2025 Aug 9;32(5):dsaf020. doi: 10.1093/dnares/dsaf020 (PMC12454935; doi:10.1093/dnares/dsaf020)
Supplement: dsaf020_suppl_Supplementary_Figure_1 [file dsaf020_suppl_supplementary_figure_1.docx]

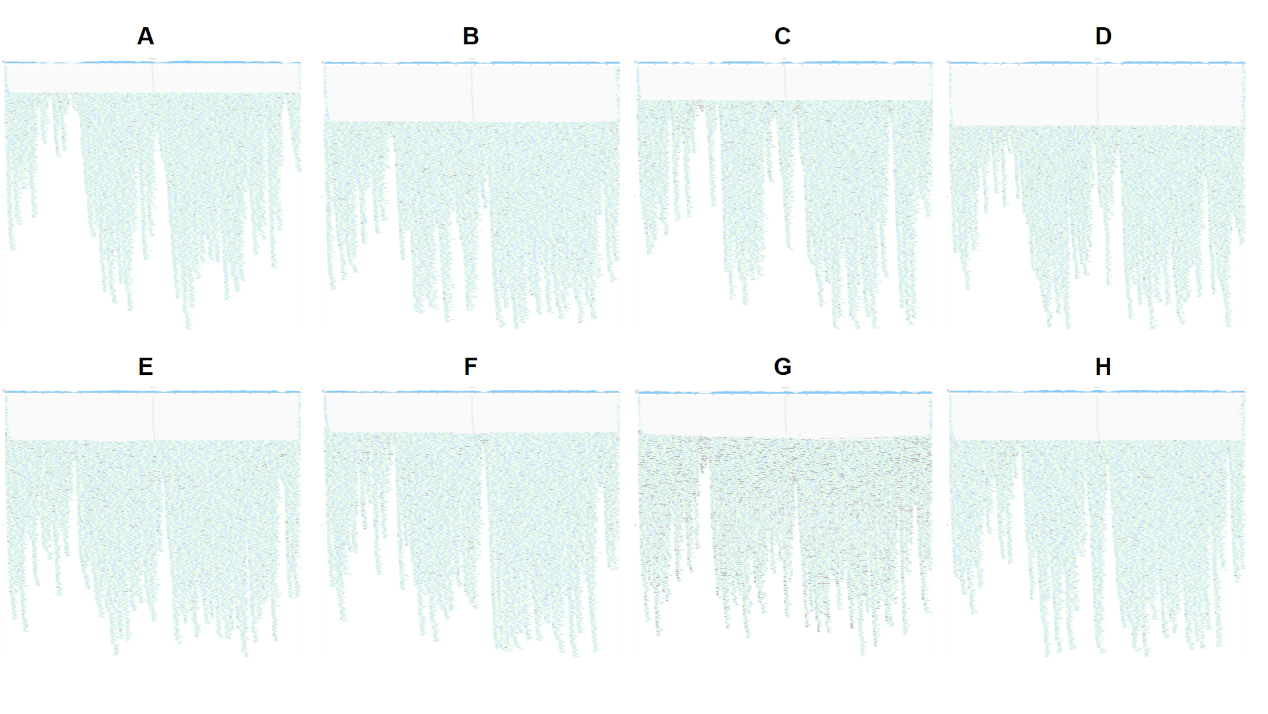


Supplementary Figure 1. Clean reads coverage and depth for mitochondrial genomes of eight *Lamprotula* species. A: *Lamprotula rochechouarti.* B: *Lamprotula tortuosa.* C: *Lamprotula leai.* D: *Lamprotula caveata.* E: *Lamprotula scripta.* F: *Lamprotula zonata.* G: *Lamprotula fibrosa.* H: *Lamprotula polysticta.*


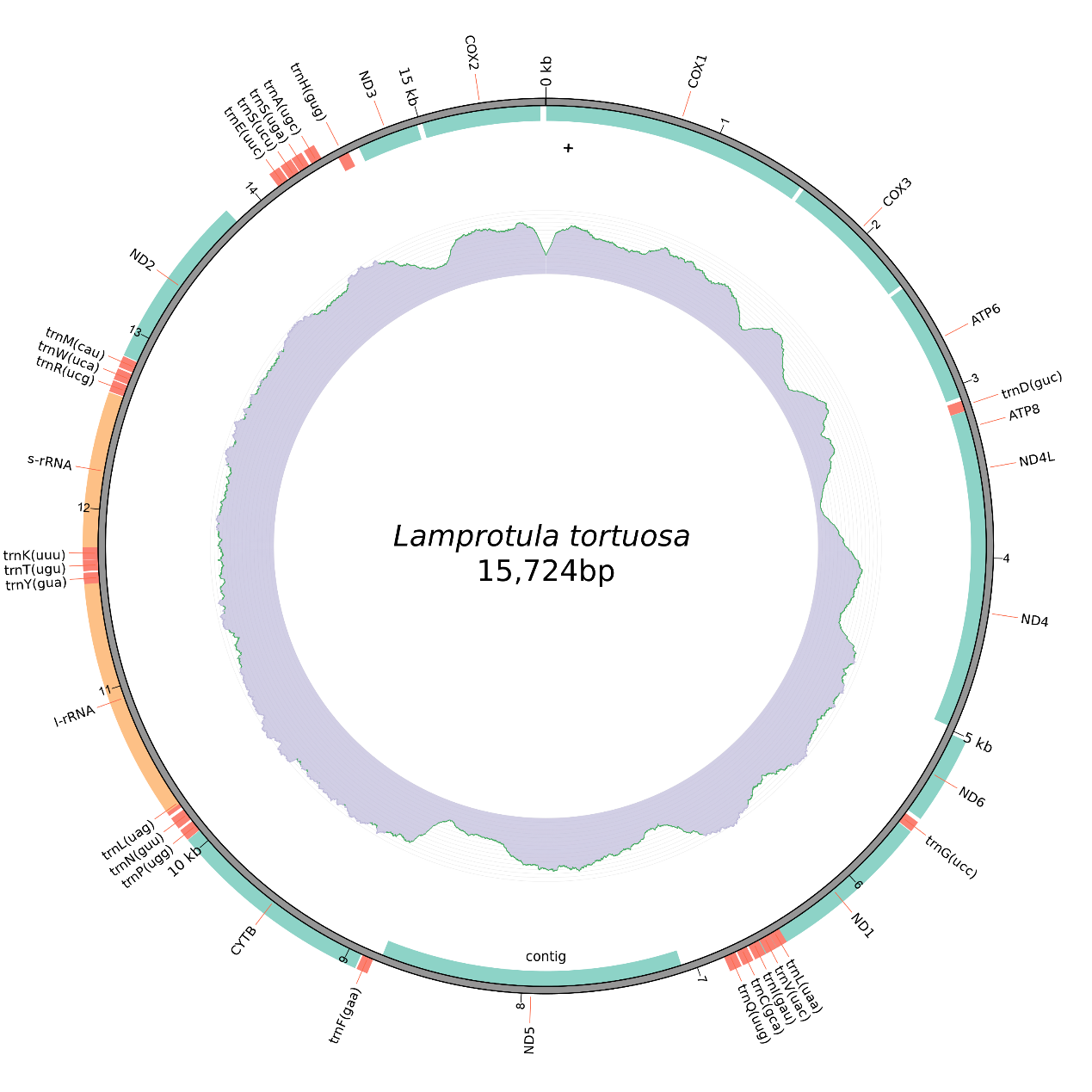


Supplementary Figure 2. Mitochondrial genome structure of *Lamprotula tortuosa*.


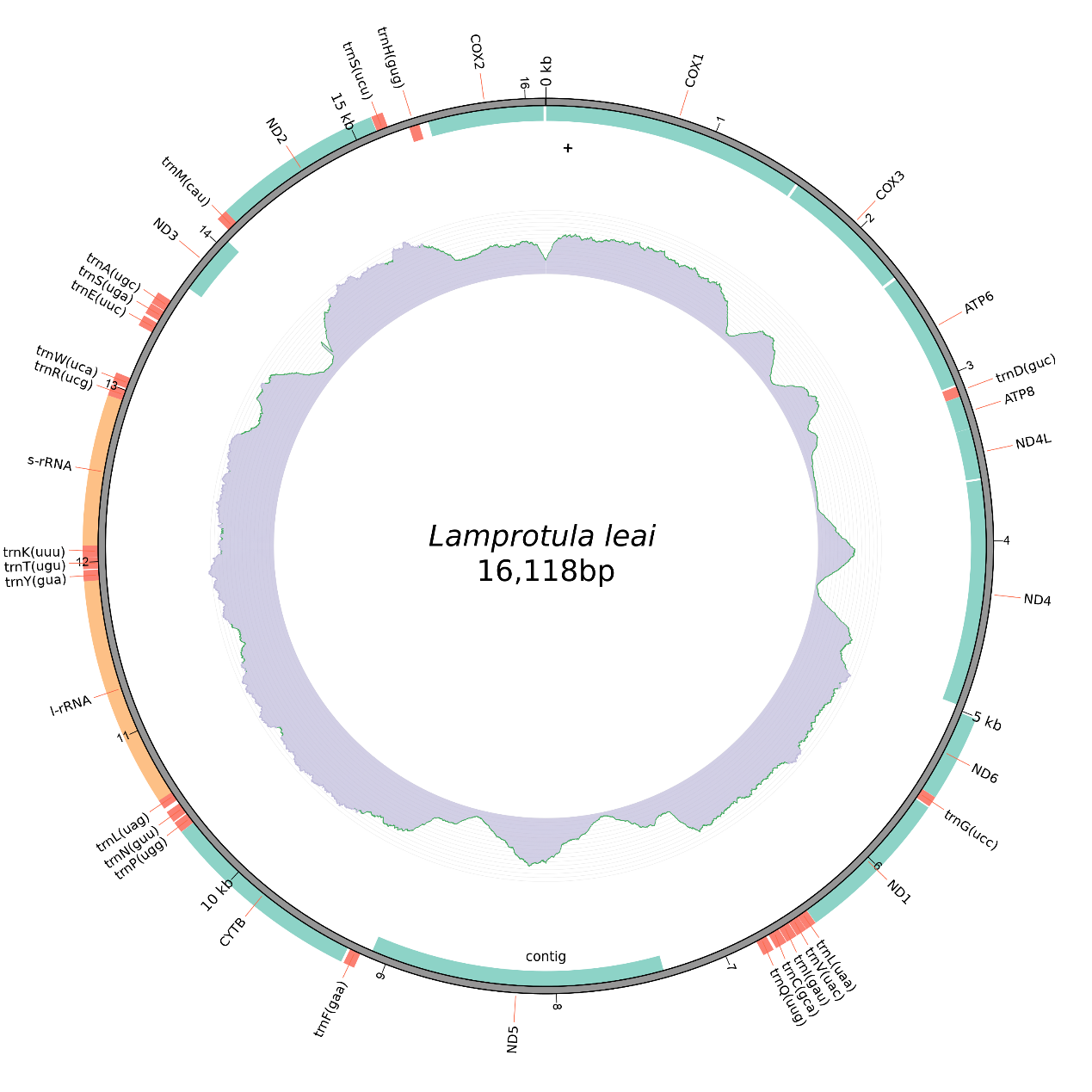


Supplementary Figure 3. Mitochondrial genome structure of *Lamprotula leai*.


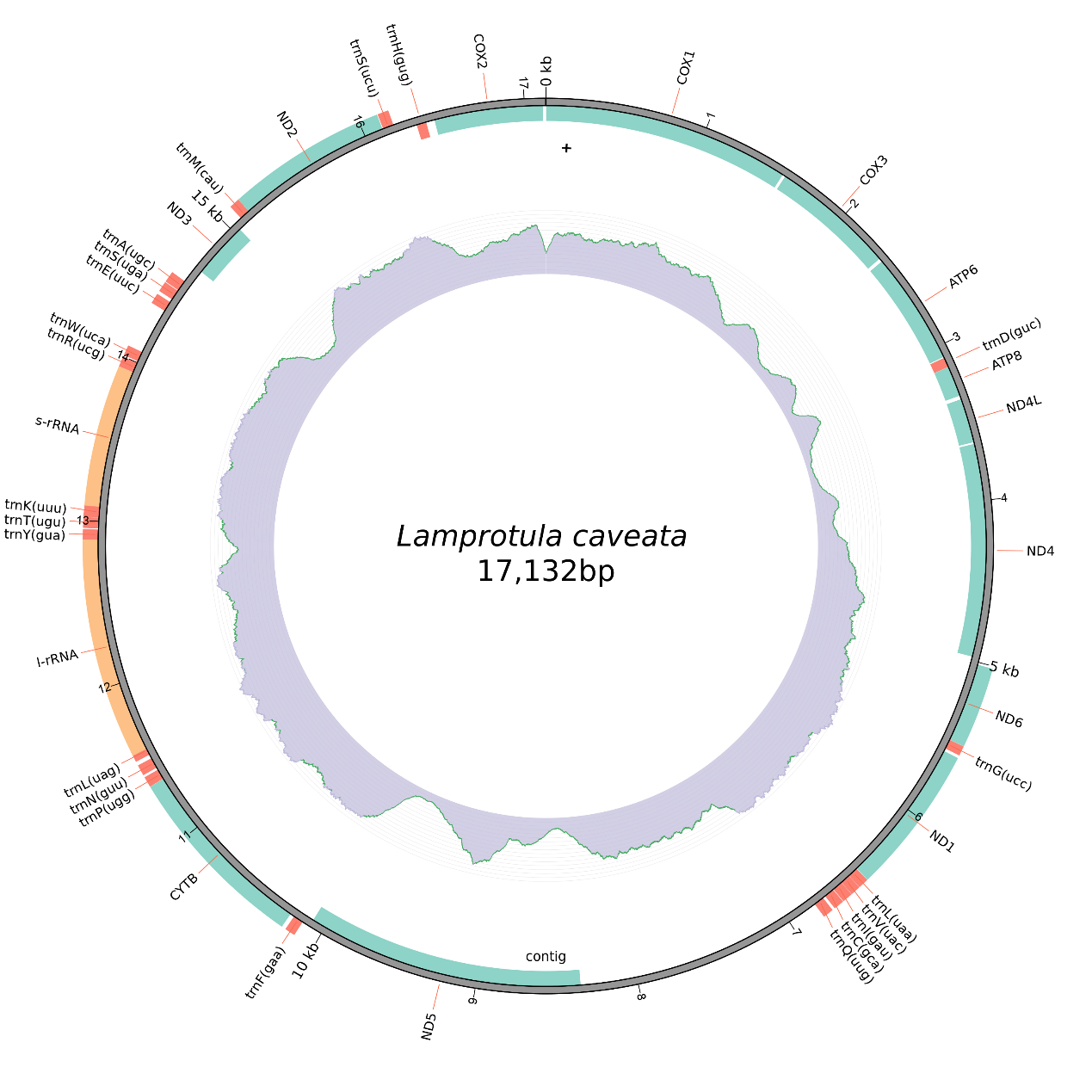


Supplementary Figure 4. Mitochondrial genome structure of *Lamprotula caveata*.


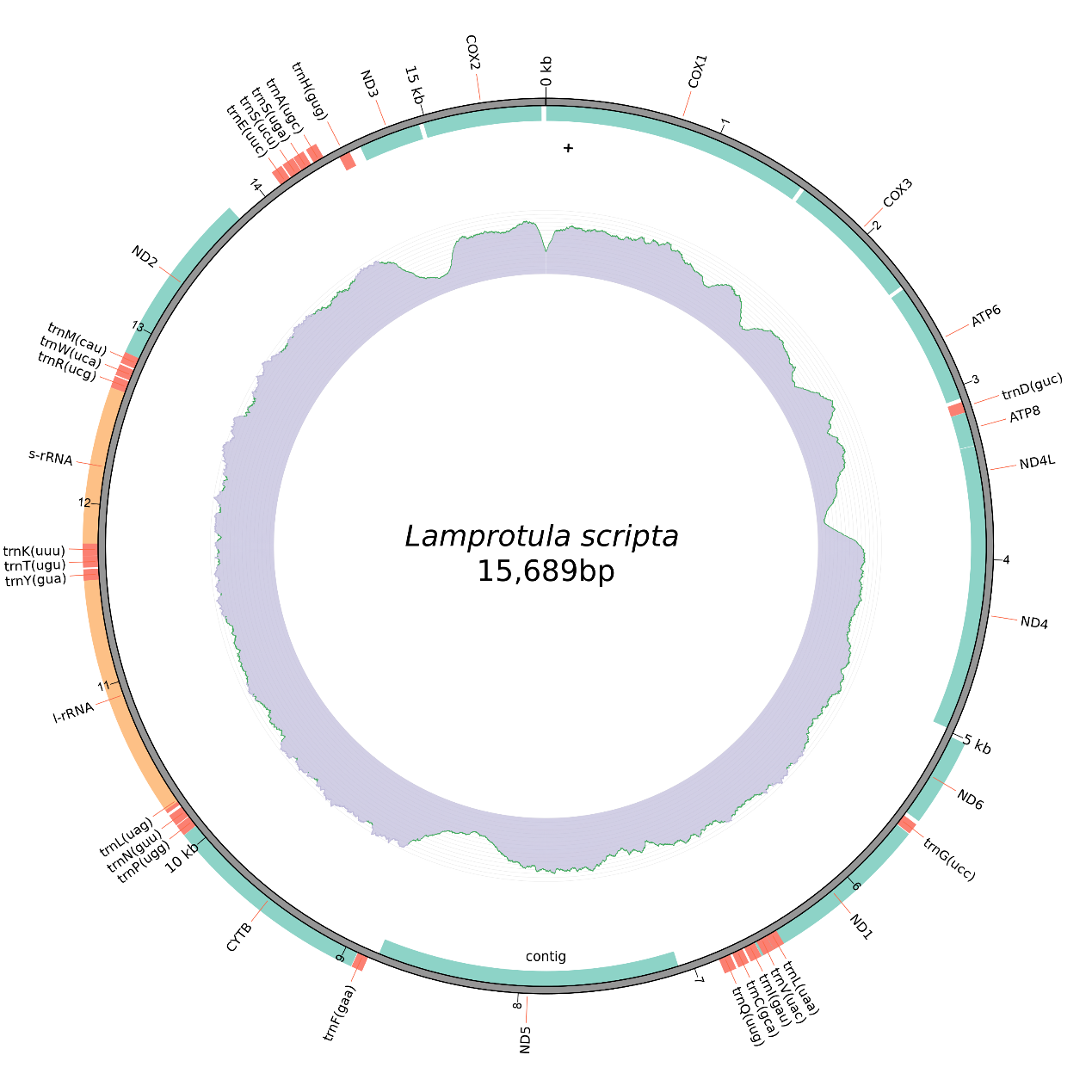


Supplementary Figure 5. Mitochondrial genome structure of *Lamprotula scripta*.


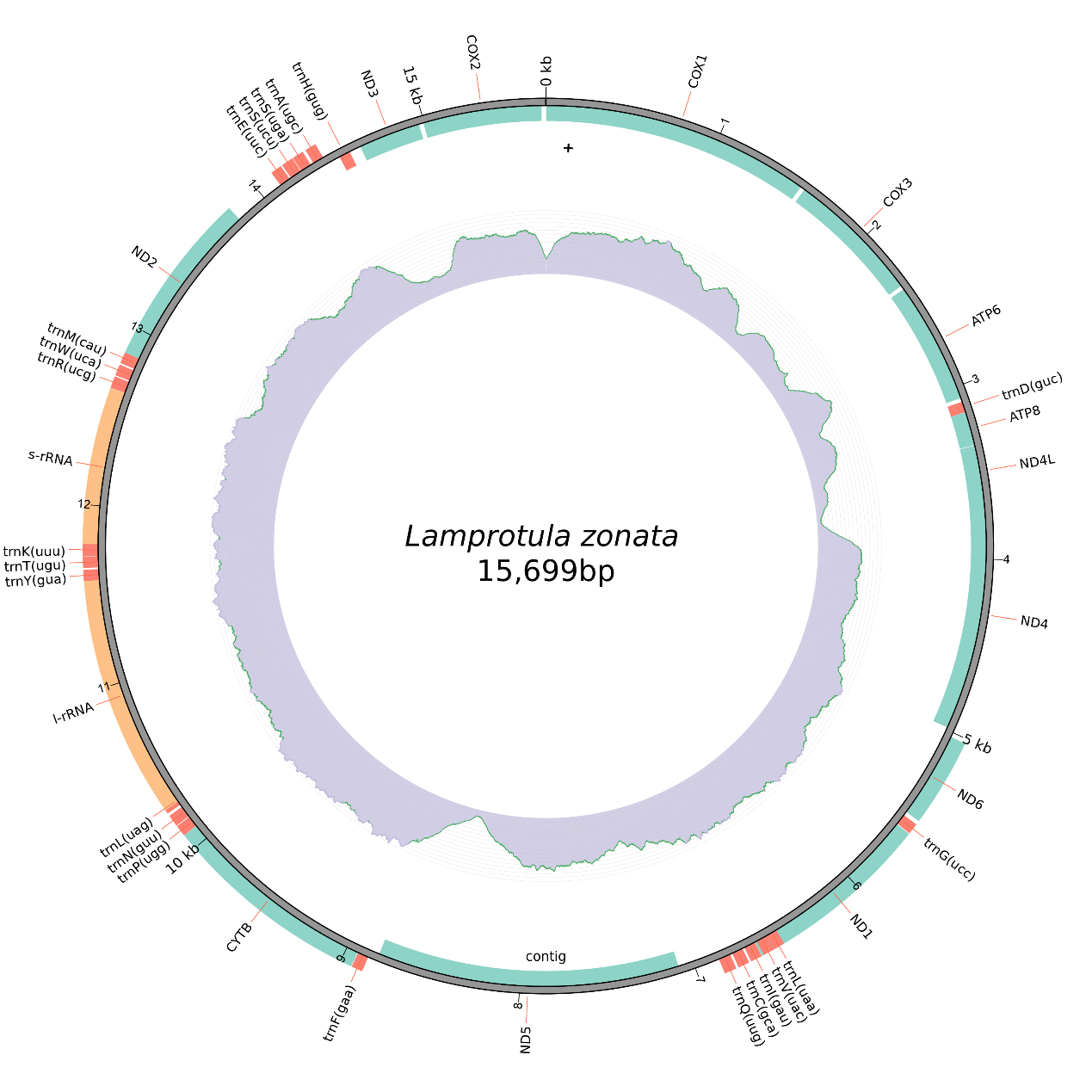


Supplementary Figure 6. Mitochondrial genome structure of *Lamprotula zonata*.


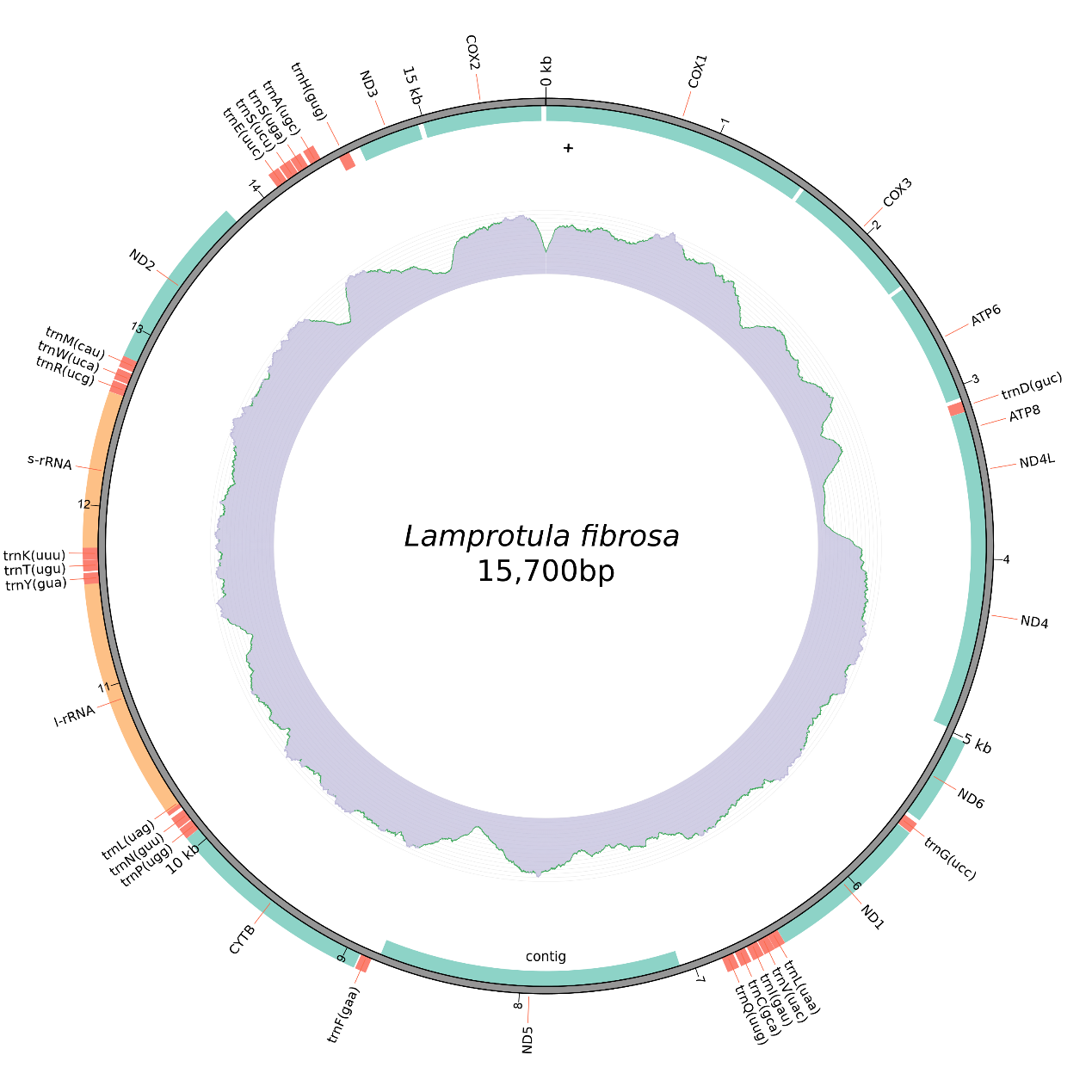


Supplementary Figure 7. Mitochondrial genome structure of *Lamprotula fibrosa*.


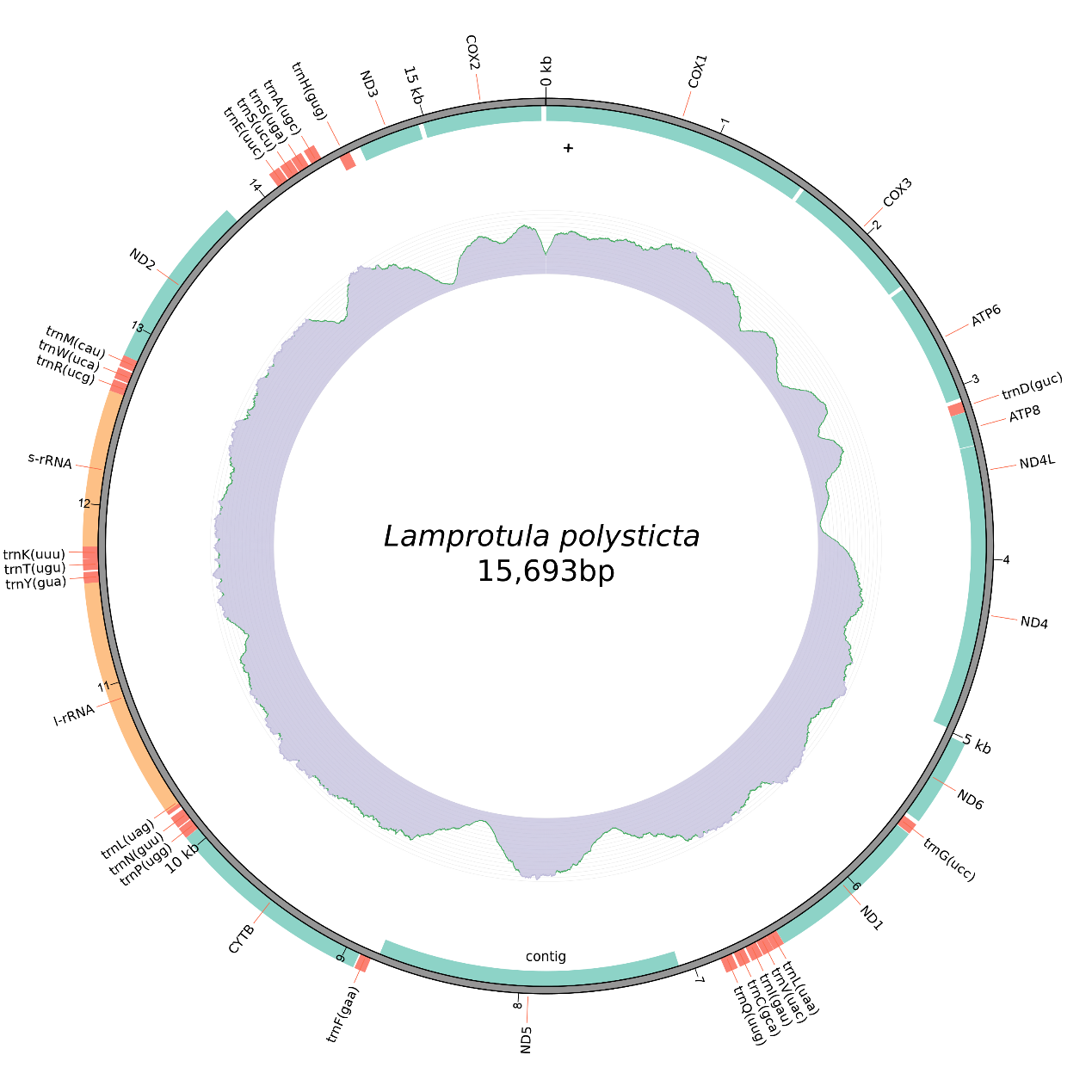


Supplementary Figure 8. Mitochondrial genome structure of *Lamprotula polysticta*.
